# Evidence-based practice among mental health professionals in Puerto Rico: Attitudes, knowledge, resources, and treatments

**DOI:** 10.1371/journal.pmen.0000103

**Published:** 2025-04-02

**Authors:** Natalia Giraldo-Santiago, Cynthia M. Navarro Flores, Alejandro L. Vázquez, Paul Fericelli-Castillo, Yoojee Kim, Natalie Rodriguez-Quintana, Alisha Vazquez, Melanie M. Domenech Rodríguez

**Affiliations:** 1 Department of Psychiatry, Massachusetts General Hospital, Boston, Massachusetts, United States of America; 2 Harvard Medical School, Harvard University, Boston, Massachusetts, United States of America; 3 Department of Psychology, University of Tennessee, Knoxville, Tennessee, United States of America; 4 Department of Social Work, Inter American University, San Juan, Puerto RicoUnited States of America; 5 TRAILS, A Project of Tides Center, San Francisco, Michigan, United States of America; 6 Department of Psychiatry, Michigan Medicine, Ann Arbor, MI, Michigan, United States of America; 7 Department of Psychology, Utah State University, Logan, Utah, Utah, United States of America; PLOS: Public Library of Science, UNITED KINGDOM OF GREAT BRITAIN AND NORTHERN IRELAND

## Abstract

The growing prevalence of mental health disorders in Puerto Rico necessitates the use of efficacious mental health interventions, such as Evidence-Based Treatments (EBTs), to promote positive outcomes within this underserved population. This study aimed to describe the use of psychosocial interventions with different levels of evidence for efficacy (i.e., well-established, probably or possibly efficacious, and experimental) among mental health professionals in Puerto Rico (i.e., social workers, psychologists, counselors; *N* = 237). A quantitative cross-sectional design was employed to analyze data collected between October and December 2021. Chi-square tests of independence were conducted to examine differences in the self-reported use of interventions across professional disciplines and agency types. A significant portion of the sample (71.7%) reported using at least one intervention with well-established evidence. Psychologists most frequently used these well-established interventions in their practice, followed by counselors and social workers. Participants working in private agencies also endorsed higher utilization of various EBTs than those in public agencies. Logistic regression analyses were performed to assess the correlation between favorable attitudes, knowledge, and training on these practices and the self-reported use of specific interventions. Favorable attitudes towards Evidence-Based Practices (EBPs) were related to increased use of mindfulness therapy, a well-established intervention. Greater knowledge and training on EBTs were found to correlate with self-reported use of interventions with well-established and probably efficacious evidence. Findings suggest that mental health professionals in Puerto Rico use some well-established EBTs, but the modalities used varied based on a variety of factors. Our findings suggest that increasing knowledge and agency training on EBTs may be an effective way to promote the use of interventions with well-established and probably efficacious evidence. Implications for research, practice, education, and policy development in Puerto Rico are discussed.

## Introduction

Puerto Ricans residing on the United States mainland and the island experience high rates of mental health problems due to social marginalization and the effects of colonization (i.e., most frequently being diagnosed with major depression, anxiety, substance‑related) [1–4]. Puerto Ricans also face various obstacles to accessing mental health services across both contexts [3,5]. When they do successfully navigate the myriads of barriers impeding their access to needed mental health services (e.g., affordability, availability, stigma), they rarely receive high-quality evidence-based and/or culturally competent/adapted care [5]. Efforts have been made to address various mental and behavioral health problems through increased dissemination of accessible and culturally adapted evidence-based treatments (EBTs) for the Hispanic and Latinx population [6–8]. For example, the Substance Abuse and Mental Health Service Administration (SAMHSA) launched an online platform of empirically supported interventions and practices, and more recently, resources have been distributed to guide the adaptation of EBTs for under-resourced and diverse populations [9,10]. While many mental health professionals have culturally adapted a number of EBTs originally developed for White and affluent populations, their accessibility and effectiveness for the Latinx population remains limited [11–16]. Further efforts are needed within the Puerto Rican context to increase access to effective EBT.

In Puerto Rico, interventions with insufficient evidence may be used in clinical settings due to limited access to those that are culturally adapted and proven to be effective. A recent study found that some mental health professionals felt EBTs were a good resource but may not be suitable for Puerto Ricans without proper adaptations, as they currently lack rigorous clinical trials in this population [17]. Further, professionals have expressed hesitancy to adopt these practices due to time constraints and perceived lack of availability of resources in Spanish [17]. Another possible reason for the continued use of non-EBTs in Puerto Rico might be that the Code of Mental Health Services in Puerto Rico (i.e., *Ley de Salud Mental de Puerto Rico* # 408 [Law of Mental Health of Puerto Rico]) acknowledges the use of faith-based and pastoral counseling services for the treatment of addiction in community-based and non-profit private organizations [18], as these could be culturally and linguistically congruent with the context and values of Puerto Ricans [19,20]. However, research has yet to examine EBT utilization patterns among mental health practitioners within Puerto Rico. A better understanding of the mental health interventions utilized by mental health practitioners in Puerto Rico could help identify gaps and areas of opportunity to move toward the adaptation of EBTs for this clinically underserved group. This is particularly important, as evidenced by the limited literature on Latinx needs and challenges accessing culturally adapted psychosocial interventions [21].

Dissemination and implementation science commonly focuses on attitudes, needs, and knowledge as methods of promoting the adoption and sustainment of broader Evidence-Based Practices (EBPs). EBPs represent the process of integrating clinical experience, patient preferences and needs, and the best available evidence-based mental health services to promote positive treatment outcomes [21,22]. These elements also align with the Theory of Planned Behavior (TPB), which explains how behavioral attitudes, subjective norms, and perceived behavioral control influence a person’s intention and subsequent engagement in a particular behavior [23]. The TBP framework provides theoretical guides on factors that may influence clinicians’ engagement in behaviors such as utilizing EBTs. The present study considers the TBP framework to understand factors associated with Puerto Rican mental health service practitioners’ attitudes (e.g., views towards EBTs), subjective norms (e.g., the type of organization providers work at, the mental health professional field), and perceived behavioral control over the use of EBTs (e.g., knowledge of EBTs and trainings which can increase self-efficacy in EBTs).

Research on the implementation of EBPs has found the factors within the TBP model to be important in mental health providers’ utilization of EBTs and EBP. For example, in Puerto Rico, two quantitative studies found that mental health providers had favorable attitudes toward the adoption of EBPs [24], with younger practitioners, those with greater educational attainment (i.e., doctoral degree), and those with more knowledge of EBPs being more involved in EBP behaviors [25]. Further, other studies have found changes in EBT attitudes and competence following the completion of specialized training programs [25] or because of social influences and norms established within a specific agency, profession, and/or professional group towards the use of EBTs [26,27]. However, less attention has been given to the self-reported use of EBTs, which consists of mental health providers’ preference toward a specific treatment modality. In this study, we differentiate the process of using EBPs and EBTs, as the latter informs a specific psychotherapeutic approach that an individual professional chooses to incorporate into their practice (e.g., Cognitive Behavioral Therapy as the preferred EBT). Learning more about the interventions used by Puerto Rican mental health practitioners could help provide information about emerging trends and related correlates for promoting EBTs within this context.

### The present study

This study examined self-reported use of psychosocial interventions with different levels of evidence among an interdisciplinary group of mental health professionals in Puerto Rico. Interventions were categorized by their scientific rating level based on methods criteria requiring a) randomized controlled designs, b) treatment manuals or logical equivalents, c) specified target populations, d) reliable and valid outcome measures related to the targeted problem(s), and e) appropriate statistical analyses with sufficient sample sizes [28,29]. Ratings ranged from well-established evidence of efficacy (Level 1), probably efficacious interventions supported by research (Level 2), possibly efficacious interventions (Level 3), and indications that there is promising research for interventions not yet tested in a randomized control trial or those tested in at least one clinical study but without sufficient evidence to meet level 3 criteria (Level 4). No intervention was designated to the questionable efficacy category, as none were found to be inferior to other treatment groups and/or control groups (level 5; 28). We also assessed differences across disciplines (i.e., social workers, psychologists, and counselors) and agency type (public vs. private), as well as how individual characteristics (e.g., attitudes and knowledge related to EBT adoption) and organizational factors (e.g., training received) related to self-reported use of each treatment across the four different levels of efficacy.

We hypothesized that there would be differences in the use of EBTs by agency type (i.e., public vs. private institutions) and occupation (i.e., psychologists vs. other professionals). We also hypothesized that experimental approaches and practices with less scientific evidence (Level 4) would be more likely to be utilized in private agencies, as suggested by the Law of Mental Health of Puerto Rico and the existing literature [21,25], and that providers in public settings would be less likely to use practices with well-established evidence (Level 1). We also hypothesized that psychologists would be more inclined to use EBTs with well-established evidence when compared to social workers and counselors, and that favorable attitudes, greater knowledge, and EBT training would be predictors of the use of treatments with well-established evidence (level 1).

## Materials and methods

### Study design and setting

A quantitative cross-sectional approach was used to collect in-depth survey data from mental health professionals. A self-administered survey was used to gather data from practicing and licensed social workers, counselors, and psychologists in Puerto Rico. Data was collected online and in-person between October and December of 2021, including data collection conducted at an in-person professional conference. Participants could choose to participate in English or Spanish. The Qualtrics online survey platform was used to collect data from participants that wished to complete the survey electronically. Participants at the conference were given the option to complete a pen and paper survey or scan a quick-response (QR) code to complete the Qualtrics survey. Participants were not asked to specify how they learned about the study (i.e., in-person or online), so recruitment sources were not tracked. Data collection efforts occurred through an anonymous approach to allow participants to feel comfortable answering questions related to their demographics and attitudes toward EBPs and treatment modalities.

### Study population and recruitment

The target population of the present study consisted of practicing and licensed social workers, psychologists, and mental health counselors in Puerto Rico. This sample of mental health professionals was recruited through e-mail correspondence via the following associations: (a) Professional Counselors Association (CPCR), (b) Psychology Association of Puerto Rico (APPR), and (c) College of Social Work Professionals (CPTSPR). Social workers were also recruited at an in-person conference hosted by CPTSPR in Ponce, Puerto Rico in October 2021. Participants had to reside in Puerto Rico; identify as Latinx (e.g., Puerto Rican, Cuban, Dominican); be licensed to practice social work, counseling, or psychology; and be employed part-time or full-time at a public or private agency in Puerto Rico. Psychiatrists were not recruited as they primarily focus on medication management as a treatment for mental health difficulties rather than providing psychotherapy (U.S. & Puerto Rico).

### Data collection

The survey took approximately 10–15 minutes to complete. Participants who responded affirmatively to the screening questions that confirmed their eligibility for the study proceeded to the informed consent form. The consent form appeared before the start of the survey and discussed the benefits and risks of participating in the study. Information about the principal investigator was available if questions or concerns arose at any point throughout the survey process. Those who provided informed consent were administered the survey. Survey responses were stored in a password-protected platform and the data is expected to be deleted after a five-year period. The paper-based responses were double entered into Qualtrics to combine responses from both recruitment sources and to prevent data entry errors.

### Study variables

#### Outcome variables.

A list of 18 psychosocial interventions with varying degrees of evidence were presented to participants to indicate their use (if any). Consistent with the American Psychological Association’s efforts to promote and disseminate strong psychological procedures [29], a five-level ranking system was used to determine intervention efficacy and potency. The scientific rating/ranking assigned to each treatment was determined based on available clearinghouses with resources for behavioral health providers including the California Evidence-Based Clearinghouse for Child Welfare, the Title IV-E Prevention Services Clearinghouse, and the Penn State Clearinghouse for Military Family Readiness [30–32]. For interventions not found in the clearinghouses, systematic reviews, randomized control trials (RCTs), meta-analysis, case studies, or other resources (brief overviews) were utilized to categorize them in their corresponding level based on their efficacy. The evaluation criteria was broken down into five levels: Level 1: well-established evidence of efficacy (at least two independent randomized controlled trials (RCTs) showing the treatment is superior to a placebo or equivalent to another well-established treatment), Level 2: probably efficacious (at least two RCTs demonstrating superiority to a waitlist control or one good experiment meeting well-established criteria [level 1] but lacking independent replication), Level 3: possibly efficacious interventions (at least one randomized control trial [RCT] showing the treatment is better than a waitlist or control group, or two or more studies meeting most methodological criteria but are not RCTs), Level 4: experimental (interventions not yet tested in a RCT or lacking sufficient evidence to meet level 3 criteria), and Level 5: questionable efficacy (treatments tested in quality studies that were shown to be ineffective or inferior, with no evidence of beneficial effects [25]. Further details on the intervention criteria can be found in the primary study [21]. No interventions fell in the questionable efficacy category, as none were found to be inferior to other treatment groups and/or control groups (level 5) [28].

It is important to note that when discrepancies in the scientific rating of interventions were found between clearinghouses, the research team categorized the intervention based on the clearinghouse that provided the highest level of support received (See Tables 1 and 2). For example, Dialectical Behavioral Therapy received a rating of promising research evidence (Level 3) in the California Evidence-Based Clearinghouse for Child Welfare [30] and a rating of probably efficacious treatment (Level 2) in the Penn State Clearinghouse for Military Family Readiness [32]. In this study, Dialectical Behavioral Therapy was categorized as probably efficacious under level 2.

**Table 1:** Chi-Square test of independence for each self-reported evidence-based treatments by type of agency and professional occupation (N =237).

	Agency Type				Occupation			
Evidence-Based Treatments (EBTs)	Total use (*n* = 178; 75.1%)	Private (*n* = 102; 43.4%)	Public (*n* = 135; 56.6%)	*p-value*	Social workers (*n* = 108; 45.6%) % of row/% of column*	Psychologist (*n* = 69; 29.1%) % of row/% of column *	Counselors (*n* = 60; 25.1%) % of row/% of column*	*p-value*
**Level 1: Well-established**	*n* = 170 (71.7%)							
Cognitive Behavioral Therapy1,2	131(55.3%)	73 (55.7%)	58 (44.3%)	<.001	30 (22.9%)–27.8%	62 (47.3%)–89.9%	39 (29.8%)–65.0%	<.001
Cognitive Processing Therapy 1,2	41 (17.3%)	23 (56.1%)	18 (43.9%)	.024	13 (12.0%)–12.0%	18 (26.1%)–26.1%	10 (16.7%) –16.7%	.054
Mindfulness Therapy 1,2,3	71 (30.0%)	46 (64.8%)	25 (35.2%)	<.001	11 (15.5%) –10.2%	39 (54.9%) –56.5%	21 (29.6%)–35.0%	<.001
Motivational Interviewing 1,2,3	67 (28.3%)	37(55.2%)	30 (44.8%)	.050	21(31.3%)–19.4%	23 (34.3%)–33.3%	23 (34.3%) –38.3%	.018
Accept. & Commitment Therapy 1	64 (27.0%)	40 (62.5%)	24 (37.5%)	<.001	15 (23.4%)–13.9%	32 (50.0%)–46.4%	17 (26.6%) –28.3%	<.001
Multisystemic Therapy 1	45 (19.0%)	23 (51.1%)	22 (48.9%)		26 (57.8%)–24.1%	10 (22.2%)–14.5%	9 (20.0%)–15.5%	
Solution Focused Therapy 1	67 (28.3%)	35 (52.2%)	32 (47.8%)	.019	21 (31.3%)–19.4%	23 (34.3%)–33.3%	23 (34.3%)–38.3%	.018
Rational Emotive Therapy3	54 (22.9%)	37 (68.5%)	17 (31.5%)	<.001	7 (13.0%)–6.5%	24 (44.4%)–34.8%	23 (42.6%)–38.3%	<.001
**Level 2: Probably efficacious**	*n* = 48 (20.3%)							
Interpersonal Therapy2, 3	35 (14.8%)	23 (65.7%)	12 (34.3%)	.012	9 (25.7%)–8.3%	18 (51.4%)–26.1%	8 (22.9%) –13.3%	.005
Dialectical Behavioral Therapy 1,2	24 (10.1%)	19 (79.2%)	5 (20.8%)	<.001	4 (16.7%)–3.7%	11 (45.8%)–15.9%	9 (37.5%) –15.0%	.011
**Level 3: Possibly efficacious**	*n* = 103 (43.5%)							
Twelve-step Facilitation4	14 (5.9%)	8 (57.1%)	6 (42.9%)		5 (35.7%)–35.7%	5 (35.7%)–35.7%	4 (28.6%)–28.6%	
Psychodynamic Therapy1	21 (8.8%)	10 (47.6%)	11 (52.4%)		4 (19.0%)–3.7%	10 (47.6%)–14.5%	7 (33.3%)–11.7%	.033
Psychoanalytic Therapy4	11 (4.7%)	7 (63.6%)	4 (36.4%)		6 (54.5%) –5.6%	1 (9.1%)–1.4%	4 (36.4%)–6.7%	
Client (Person)-centered Therapy3	87 (36.7%)	49 (56.3%)	38 (43.7%)	.015	26 (29.9%) –24.1%	28 (32.2%)–40.6%	33 (37.9%)–55.0%	<.001
	*n* = 65 (27.4%)							
Faith-based Therapy5	27 (11.4%)	17 (63.0%)	10 (37.0%)	.060	3 (11.1%)–2.8%	9 (33.3%)–13.0%	15 (55.6%)–25.0%	<.001
Creative Arts Therapy5	30 (12.7%)	19 (63.3%)	11 (36.7%)	.041	4 (13.3%)–3.7%	18 (60.0%)–26.1%	8 (26.7%)–13.3%	<.001
Abstinence-based Interventions 5	12 (5.1%)	6 (50.0%)	6 (50.0%)		7 (58.3%)–6.5%	2 (16.7%)–2.9%	3 (25.0%) –5.0%	
Existential Therapy5	23 (9.7%)	14 (60.9%)	9 (39.1%)		3 (13.0%)–2.8%	8 (34.8%)–11.6%	12 (52.2%)–20.0%	<.001

Note. Percentages only show those endorsed using the intervention (yes) within agency type and professional occupation. Frequency is displayed by row on the variable of agency type.

* Row and column percentages are shown on the occupation variable.

^1^California Evidence-Based Clearinghouse for Child Welfare, 2023

^2^Penn State Clearinghouse for Military Families Readiness

^3^Title IV-E Prevention Services Clearinghouse

^4^Systematic and meta-analysis, including the Cochrane Database

^5^Other peer reviewed sources (see references 33-39)

**Table 2:** Logistic regression analysis of attitudes, knowledge, and resources on each self-reported evidence-based treatments (n = 237).

Evidence-Based Treatments (EBTs)	Attitudes towards EBPs OR (95% CI)	*p*-value	Knowledge EBP resources OR (95% CI) ^a^	*p*-value	Training in the last year OR (95% CI) ^a^	*p*-value
**Level 1: Well-established**						
Cognitive Behavioral Therapy	1.17 (0.70–1.95)		1.77 (1.06–2.98)	.030	1.94 (1.15–3.26)	.013
Cognitive Processing Therapy	0.64 (0.32–1.27)		1.39 (0.70–2.73)		1.64 (0.81–3.31)	
Mindfulness Therapy	2.35 (1.32–4.20)	.004	2.69 (1.50–4.80)	<.001	2.61 (1.43–4.76)	.002
Motivational Interviewing	1.51 (0.86–2.67)		2.32 (1.30–4.17)	.005	1.46 (0.82–2.60)	
Acceptance and Commitment Therapy	1.08 (0.61–1.91)		2.74 (1.50–5.00)	<.001	3.11 (1.64–5.89)	.001
Multisystemic Therapy	0.88 (0.46–1.69)		1.36 (0.71–2.61)		1.73 (0.88–3.42)	
Solution Focused Therapy	1.52 (0.86–2.68)		2.54 (1.41–4.58)	.002	1.59 (0.89–2.85)	
Rational Emotive Therapy	1.23 (0.67–2.26)		1.66 (0.90–3.07)		1.29 (0.69–2.40)	
**Level 2: Probably efficacious**						
Interpersonal Therapy	0.88 (0.95–1.04)		4.16 (1.80–9.61)	<.001	3.66 (1.53–8.76)	.004
Dialectical Behavioral Therapy	0.52 (0.22–1.23)		5.98 (1.97–18.10)	.002	10.11 (2.32–44.10)	.002
**Level 3: Possibly efficacious**						
Twelve–step Facilitation	1.10 (0.38–3.24)		2.71 (0.83–8.90)		5.06 (1.11–23.13)	
Psychodynamic Therapy	0.94 (0.39–2.31)		4.93 (1.61–15.13)	.005	2.08 (0.78–5.55)	
Psychoanalytic Therapy	1.40 (0.42–4.70)		1.83 (0.52–6.42)		0.64 (0.19–2.17)	
Client(Person)–centered Therapy	1.06 (0.63–1.80)		1.25 (0.74–2.12)		1.47 (0.86–2.52)	
**Level 4: Experimental**						
Faith–based Therapy	0.91 (0.41–2.03)		2.71 (1.14–6.48)	.024	2.45 (1.00–6.05)	
Creative Arts Therapy	1.99 (0.92–4.31)		1.92 (0.87–4.24)		1.98 (0.87–4.53)	
Abstinence–based Interventions	1.55 (0.49–4.93)		2.13 (0.62–7.27)		1.60 (0.47–5.47)	
Existential Therapy	1.94 (0.82–4.63)		2.04 (0.83–5.02)		1.91 (0.76–4.84)	

^a^OR = odds ratio; 95% CI = 95% confidence interval. Attitudes were examined as a continues variables while knowledge on how to access EBP resources was examined as a categorical yes (1) or no (0). All practices were examined as a self–reported intervention.

Interventions not available in clearinghouses were referenced in the research literature to determine their categorization. For instance, Rational Emotive Therapy was not referenced in clearinghouses but categorized under the well-established evidence (Level 1) given the extensive literature supporting this psychological intervention [33,34]. Interventions not yet tested in a randomized controlled trial and/or tested in at least one clinical study but that were not sufficient to meet level 3 criteria were categorized as experimental (level 4). This was the case of Faith-based Interventions [35], Creative Arts Therapies [36], Abstinence-based Interventions [37,38], and Existential Therapy [39]. See Table 1 for a list of interventions separated into the four categories of evidence. Additionally, an open-ended question for participants to add any other intervention not included in the list was presented.

#### Predictor variables.

Individual and organizational factors were examined to explore their relationships with the self-reported use of these practices. Knowledge and attitudes related to EBTs are considered important individual factors for the adoption of empirically supported interventions. Meanwhile, agency type (public vs. private) and EBT training were considered organizational correlates of self-reported interventions. Details on each measure are presented below. The Evidence-Based Practice Attitude Scale (EBPAS-15) [40] measures favorable attitudes toward EBPs. The scale has 15 items calculated as a mean of the total after reverse scoring adjustments. Participants rate items on a 5-point Likert scale ranging from *not at all* (1) to a *very great extent* (5). The EBPAS has been found to have acceptable reliability (α =.77) with a Spanish sample [41]. In this sample, the reliability of the scale was excellent (α =.89) [42].

A single item, created for this study, asked practitioners whether they had knowledge of how to access evidence-based psychotherapy resources. Respondents reported *yes* (1) or *no* (0).

Participants were asked whether they were *social workers* (1), *psychologists* (2), or *counselors* (3) on a single item. Participants were asked to answer the following question: “*What type of agency do you provide services?”*. Responses ranged from *public* (1), *private for-profit* (2), *private non-profit community-based agency* (3), and *private practice* (4). This variable was recorded as *public* (1) and *private* (0) to provide sufficient representation to perform statistical analyses. One question examined whether the participants had received EBP training in the previous year. Responses were *yes* (1), *no* (0), or *I do not know* (2). Responses with *I don’t know* were coded as *no* (0) to make responses dichotomous.

#### Statistical data analysis.

Data analyses for this study were conducted on March 22, 2023. SPSS IBM Corp 2021 version 28 [43] was used to perform data analyses. Descriptive statistics were used to characterize the demographics of the sample, as well as their reported use of EBTs. Chi-square tests of independence were used to examine differences in the use of EBTs across professional disciplines and agency types. Variable frequency and percentages of EBTs self-reported by participants based on their agency and occupation were explored. Logistic regression models were then used to examine whether knowledge, attitudes, and training received in the past year predicted the self-reported usage of EBTs among Puerto Rican mental health professionals. Authors did not have access to information that could identify individual participants during or after data collection. Sample size was determined by conducting a G*Power 3.1 analysis, with a desire power of 0.8 and tolerance for Type I error (α) set to.05. It was estimated that a sample of 120 participants was needed to achieve adequate statistical power.

#### Data management and quality control.

A total of 299 individuals completed the survey. Survey responses from 62 participants were removed from the analysis as the survey completion time was less than five minutes or the survey was completed by individuals living outside of Puerto Rico. Information about internet protocol (IP) addresses and location of responses were provided by the Qualtrics platform and were used to ensure responses were from within Puerto Rico.

#### Ethics statement.

This study was approved by the University of Houston Institutional Review Board (IRB approval number: STUDY00003272) on October 1, 2021. All participants provided written informed consent before taking part in the study. Steps to ensure anonymous responses were discussed in the consent form, as the researcher asked general demographic information and questions specific to their work. No identifiable personal or agency information was gathered in the survey. A $5 gift card was offered as an incentive for the time/participation of this hard-to-reach sample of Latinx mental health professionals [44].

## Results

### Participants

The total sample for analysis included 237 participants. The sample identified predominately as female (83.4%) and Puerto Rican (96.2%), with a mean age of 42.83 (*SD* = 11.64). Almost half of the participants were social workers (*n* = 108, 45.6%), followed by psychologists (*n* = 69, 29.1%) and counselors (*n* = 60, 25.1%). More than half of the respondents worked in public (*n* = 135, 56.6%) versus private (*n* = 102, 43.4%) agencies, which included for-profit, nonprofit community-based agencies, and those in private practice. In the current sample, 38.8% (*n* = 92) of the providers worked with children and/or young adults, 32.6% (*n* = 75) reported working with adults and/or older adults, and 29.5% (*n* = 70) reported working with people of all ages. All participants completed their education in Puerto Rico and a majority graduated from a private institution (82.0%). More details about the sample can be found in the main publication [21].

### Self-reported treatments by type of agency and professional occupation

Three-quarters of the sample of mental health professionals (*n* = 178; 75.1%) selected at least one of the 18 interventions (Table 1) and the remaining quarter (*n* = 59; 24.9%) did not endorse the use of any of these treatments. Among those who did not select any treatment, a majority identified as social workers (*n* = 48, 20.3%), along with a few counselors (*n* = 9, 3.8%) and psychologists (*n* = 2, 0.8%). Table 1 presents the results of the chi-square test of independence, where self-reported practices were categorized under well-established evidence (Level 1), probably efficacious (Level 2), possibly efficacious (Level 3) and experimental (Level 4). Only those who endorsed using the treatment are shown. Each of the categorized self-reported interventions were examined across agency type and professional discipline. The most used treatments were Cognitive Behavioral Therapy (55.3%), Client Centered Therapy (36.7%), Mindfulness Therapy (30.0%), Motivational Interviewing (28.3%), Solution Focused Therapy (28.3%), and Acceptance and Commitment Therapy (27.0%).

### Use of treatments with well-established evidence (level 1)

Almost three-quarters of the sample (*n* = 170; 71.7%) selected at least one treatment with well-established evidence. Significant differences were found in the self-reported use of almost all interventions with well-established evidence by agency type and occupation. Overall, practitioners in private agencies reported greater use of treatments with well-established evidence as compared to those in public agencies. For example, Cognitive Behavioral Therapy was provided by 55.7% of respondents working in private agencies as compared to less than half in the public setting (44.3%), and mindfulness therapy was provided by 64.8% of respondents in private agencies as compared to only 35.2% in the public sector. Significant differences were observed across disciplines, with psychologists reporting greater use of interventions with well-established evidence, followed by counselors and social workers. For example, almost half of psychologists reported using Cognitive Behavioral Therapy (40.5%), Mindfulness (54.9%), and Acceptance and Commitment Therapy (50.0%). These percentages were significantly higher among psychologists relative to the self-reported usage of these practices when compared to social workers and counselors (See Table 1). Multisystemic Therapy (57.8%) had the highest usage rate among social workers when compared to other professionals. Some social workers used Cognitive Behavioral Therapy (28.5%), Solution Focused Therapy (31.3%), and Motivational Interviewing (31.3%). The most used interventions with well-established evidence by Puerto Rican psychologists were Mindfulness Therapy (54.9%) and Acceptance and Commitment Therapy (50.0%). For counselors, the most used interventions were Solution Focused Therapy (34.3%) and Motivational Interviewing (34.3%). Additional bi-variate analysis was conducted to examine the overall frequency of each EBTs by column (e.g., within disciplines).

### Use of treatments with probably (level 2) and possibly efficacious (level 3) evidence

Interventions with probably efficacious evidence (Level 2) were self-reported by 20.3% (*n* = 48) of the sample. These interventions included Dialectical Behavioral Therapy (10.1%) and Interpersonal Therapy (14.8%). Interventions in Level 3 of possibly efficacious (43.5%) were more frequently reported (*n* = 103; 43.5%) than those in Level 2 (20.3%) of probably efficacious, but less likely to be reported than those under the well-established support category (Level 1; 71.7%).

Significant differences in the use of probably efficacious practices (Level 2) were found by agency and occupation. For example, Dialectical Behavioral Therapy and Interpersonal Therapy were more heavily used in private agencies than public settings. Interpersonal Therapy was more frequently reported by psychologists (51.4%) than counselors (22.9%) and social workers (25.7%; see Table 1). In the possibly efficacious practices (Level 3), Psychodynamic Therapy was used by nearly half of the sample of psychologists (47.6%) relative to social workers (19.0%) and counselors (33.3%). Meanwhile, counselors (37.9%) had greater use of possibly efficacious interventions such as Client (Person) Centered Therapy, followed by psychologists (32.2%) and social workers (29.9%).

### Use of experimental treatments (level 4)

Treatments categorized as experimental consisted of Faith-based Therapies, Creative Arts Therapy, Abstinence-based Services, and Existential Therapy. These practices were categorized as experimental due to the lack of rigorous clinical studies evaluating the effectiveness of these interventions [35–39]. Over a quarter of the sample (*n* = 65, 27.4%) selected at least one intervention classified as experimental. For example, Faith-based Therapy and Creative Arts Therapy were reported by 11.4% and 12.7% of all mental health professionals, respectively, and Abstinence-based Interventions (5.1%) were less commonly used. Significant differences were found only in the use of Creative Arts Therapy and Faith-based Therapy by agency and occupation. The chi-square test of independence neared significance (*p* =.060), with private agencies being more likely to use faith-based therapy (63.0%). Similarly, Creative Arts Therapy was significantly more used in private agencies (63.3%) when compared to public agencies (36.7%). Across occupations, Faith-based Therapy was most likely to be used by counselors (55.6%), followed by psychologists (33.3%), and a small portion of social workers (11.0%).

An open-ended question for participants to indicate any other interventions not included in the list revealed that one person reported using “*aromatherapy, auriculotherapy”,* two using *“the trans-theoretical model for addictions*”, and another two individuals reported using “*play therapy”* and *“eye movement desensitization and reprocessing (EMDR)*.”

### Knowledge, training, and attitudes on well-established treatments (level 1)

Slightly over half of the sample, 133 participants (56.1%), reported being trained in EBTs in the previous year. Meanwhile, only 117 (49.4%) participants knew how to access EBP resources. The overall evidence-based attitude score on the EBPAS scale was 2.69 (*SD* = 0.05), signaling relatively favorable attitudes towards these practices. Favorable attitudes towards the adoption of EBTs was only significantly related to the self-reported use of Mindfulness Therapy (OR = 2.35, 95% CI [1.32-4.20], *p* =.004), which may be explained by a positive skew in attitudes towards EBTs within the current sample. Those with favorable attitudes towards research-informed interventions were approximately twice as likely to report the use of Mindfulness Therapy.

The remaining treatments were predicted by knowledge and training in the year prior completing the survey. Knowledge of how to access EBP resources was generally associated with higher odds of endorsing the use of most interventions with well-established evidence, with the exception of Cognitive Processing Therapy, Multisystemic Therapy, and Rational Emotive Therapy. For example, knowledge on how to access EBT resources was a significant predictor for the self-reported use of several treatments with well-established evidence of efficacy such as Cognitive Behavior Therapy (OR = 1.77, 95% CI [1.06, 2.98], *p* =.030) among other practices. Less treatment options were significantly different when considering access to training in the past year. However, training in the previous year strongly predicted Cognitive Behavior Therapy, Mindfulness Therapy, and Acceptance and Commitment Therapy usage. Mindfulness Therapy was the only intervention that was able to be predicted by attitudes, knowledge, and training in the past year.

### Attitudes, knowledge, and training on probably (level 2) and possibly efficacious (level 3) treatments

Dialectical Behavioral Therapy and Interpersonal Therapy were associated with knowledge and training in the last year. Individuals who knew how to access EBT resources and received training on EBTs in the past year were nearly six times more likely to report using Dialectical Behavioral Therapy (OR = 5.98, 95% CI [2.32, 44.10], p =.002) and ten times more likely to report using Interpersonal Therapy (OR = 3.66, 95% CI [1.53, 8.76], p =.004). Similarly, mental health providers who knew how to access EBT resources were approximately five times more likely to engage in Psychodynamic approaches (OR = 4.93, 95% CI [1.61, 15.13], p =.005). Knowledge and training were not significant predictors of any other possibly efficacious interventions.

### Training in the use of experimental interventions (level 4)

Faith-based Interventions were twice as likely to be predicted by those who had knowledge of EBP resources (OR = 2.71, 95% CI [1.14, 6.48], p =.024) and received EBP training in the last year (OR = 2.45, 95% CI [1.00, 6.05], p =.051; See Table 2).

## Discussion

A recently published study conducted with an interdisciplinary sample of Puerto Rican mental health professionals found that social workers, counselors, and psychologists showed similar interest and favorable attitudes toward EBTs [21]. Building upon this study, we sought to examine whether the use of EBTs (e.g., self–reported behaviors) varied across individual and organizational factors, such as discipline (social worker, psychology, counseling) and agency type (public, private). Moreover, we examined associations between EBP attitudes, knowledge, training, and self–reported use of treatments with well–established, probably efficacious, possibly efficacious support, and at an experimental stage.

In all, our examination of the current state and use of EBTs among Latinx mental health professionals in Puerto Rico showed a significantly high uptake of interventions with well–established evidence among mental health professionals on a broad level (Level 1). Uptake did vary by different settings and occupations, and by individual and organizational factors such as attitudes, knowledge, resources, type of agency, and occupation. Consistent with studies with large samples in the United States, significant differences in the self–reported behaviors related to EBTs were found between social workers and psychologists [45]. Although an exciting number of providers reported using EBTs, a quarter of this sample of licensed mental health professionals (24.9%) still did not select or report using any of the interventions presented. This could be explained by the fact that almost half of the providers (43.9%) were not trained on EBPs in the year prior to the survey, and only 49.4% reported having knowledge of how to access EBP–related resources, which could potentially be related to the lack of training on EBPs. Future research should explore potential barriers to engaging in EBP training and accessing EBP resources among mental health professionals, and how these factors relate to the utilization of EBTs.

Attitudes toward EBTs as measured by the EBPAS scale were largely favorable among this sample of Puerto Rican mental health professionals. Interestingly, favorable attitudes were not significantly related to specific treatments, with the exception of Mindfulness Therapy. The rapid and growing body of research (i.e., randomized clinical trials) on Mindfulness Therapy [46] and available online resources in Spanish could have resulted in its successful dissemination and adoption among mental health professionals in Puerto Rico. While the current study did not assess in detail favorable attitudes towards mindfulness and mind–body programs, future research could explore whether these attitudes are related to the practicality of implementing these interventions or whether they are perceived to be socially and culturally congruent practices when compared to other approaches. It is important to note that quantitative methods and measures used in this study are limited and undergoing psychometric testing [42], warranting the need of future studies using qualitative methods to provide in–depth understanding of this complex phenomenon.

It is important to note that Cognitive Behavioral Therapy was the most endorsed intervention within this interdisciplinary sample. This finding suggests that the gap between research and clinical practice has narrowed in Puerto Rico. For example, Cognitive Behavioral Therapy is one of few interventions with most evidence and randomized control trials showing their efficacy and their cultural adaptation among child and adult samples of Puerto Ricans [47,48]. Moreover, another significant finding of this treatment modality study showed that the majority of the sample (75.1%) reported using at least one intervention with well–established evidence (Level 1). An interesting finding was that interventions with a lower experimental level of evidence (Level 4) and those that are possibly efficacious (Level 3) were selected by 27.4% and 43.5% of the sample, which was greater than the number of people who self–reported using interventions with probably efficacious evidence (20.3%; Level 2). These findings may indicate potential gaps in treatment and/or a preference towards less scientifically supported interventions (Level 3 and 4) either because these are perceived to be more culturally congruent or because there is still limited scientific evidence and resources (clinical materials) tailored for the Puerto Rican population.

We found that the use of interventions at an experimental stage (i.e., Faith–based and Creative Arts Therapy) was more prevalent among mental health professionals in private settings than in public agencies. This might be attributed to Puerto Rico’s Code of Mental Health [25], which permits the use of traditional, historical, and faith–based interventions in community–based and non–profit private settings. Findings of this study have both implications at an organizational level as well as occupational, as significant differences were found in the use of faith–based interventions, creative arts therapy, and existential therapy across disciplines, these being more frequently used by counselors and psychologists when compared to social workers. It is possible that providing an intervention at the experimental stage (e.g., Faith–based) could also be viewed as a way to offer culturally congruent services for Puerto Ricans. In this study, the outcome variable (i.e., usage of EBTs) was presented with the chance for practitioners to select all (if any) interventions, which allowed for the selection of multiple EBT approaches. Future research could explore mental health providers’ integrative clinical patterns to better understand which EBTs are used in combination with experimental and possibly efficacious practice. Such work could inform future clinical studies as well as the development of culturally adapted approaches to better meet the needs of Puerto Ricans. For instance, it would be worth examining whether Faith–based and Creative Arts Therapy are being combined with more established evidence such as Mindfulness and Cognitive Behavioral Therapy to make interventions more culturally congruent for Puerto Ricans.

Social workers were least likely to report using treatments across the three categories of probably efficacious, possibly efficacious, and at an experimental phase. However, social workers gravitated more towards the use of cognitive behavioral therapy (28.5%) and multisystemic therapy (57.8%) compared to other disciplines. The low percentage of self–reported clinical interventions is consistent with the Board of Examiners Act Law of social work No. 249 enacted in 1940, which currently does not recognize the clinical provision of mental health services within this profession [49]. Despite these regulations established by law, this study suggests that social workers are indeed reporting the use of well–established treatment modalities, which represents a desire to improve the quality of mental health services. In fact, all social work programs in Puerto Rico have a clinical focus accredited by the Council on Social Work Education (CSWE) to address the growing mental health needs on the island [50]. However, advances in the clinical practice of social work remain a challenge as a result of diversified opinions and paradigmatic positions within the professional group [51,52], consistent with the TBP framework describing how social norms and ideals may shape attitudes towards the implementations of EBTs [20].

An interesting finding of the current study was that favorable attitudes towards evidence–based practices did not predict the self–reported use of most EBTs, and that a higher percentage of psychologists and counselors had greater use of treatments with well–established evidence. It is important to note the presence of disparities in the use of interventions with well–established evidence was observed across agency types and occupations. For example, significant differences were found in the self–reported use of EBTs across agency types (private vs. public) and occupations (e.g., social workers vs. psychologists), and those who had greater knowledge and training displayed higher use of EBTs. Consistent with the literature, exposure to training and educational opportunities among mental health professionals can shape their EBT attitudes and increase their competence [23]. Training and resources on frameworks to conduct socio–cultural adaptations of existing EBTs may facilitate the rapid adoption and implementation of these practices in settings where they are under–utilized [12].

The study of attitudes towards the adoption of EBTs has been key to understanding the barriers and facilitators of their implementation in real–world settings [11]. This research suggests that attitudes towards new and empirically supported treatments do not necessarily translate to the actual usage or adoption of these practices. Findings should be interpreted carefully, as the outcome variables measured self–reports of clinical usage of EBTs and not the quality or delivery of these interventions. One important finding was that the presence of knowledge and training in the past year were significantly related to self–reported use of EBTs with well–established evidence suggesting that organizational factors and resources are indispensable for increasing access to and engagement in these important practices.

Furthermore, it is important to acknowledge the role of culture, as well as the lack of rigorous studies conducted with the Latinx population, which may drive mental health professionals to use a wide range of treatments with little or mixed evidence [14]. The limited research on EBTs raises a reasonable concern that mental health providers may be hesitant to adopt and implement them. Future research could focus on the level of adherence to EBP principles and practices as well as the experiences implementing these practices on their day–to–day responsibilities. Similarly, it would be important to explore barriers and facilitators in the dissemination of these practices in community and clinical settings and across interdisciplinary teams (i.e., social workers, counselors, psychologists). Lastly, examining the demographic characteristics of mental health providers in relation to their use of EBTs, as well as their attitudes, knowledge, and training in EBPs, could provide valuable insight into how these individual factors influence the adoption of EBPs among mental health professionals.

The differences in the use of interventions with well–established evidence by agency type warrants the dissemination of efficacious practices across public and private agencies, and in a variety of professional disciplines. The underutilization of well–established EBTs in public settings negatively impacts the quality of services received by individuals at a social and economic disadvantage in Puerto Rico who cannot afford private services. Consistent with the literature in Puerto Rico, the present study found that policies related to mental health services may not be favoring the integration of EBTs across all sectors (public vs. private) and consequently contributing to disparities in the use of these practices [53,54].

Drawing from existing evidence and the expertise of mental health professional and individuals in Puerto Rico experiencing mental health problems, it would be worth engaging in cultural adaptations and pilot testing the feasibility and acceptability of newly developed interventions (i.e., mindfulness) to cater to the needs of this population. More importantly, efficacy and effectiveness testing as well as the examination of implementation outcomes are greatly needed to advance the delivery of interventions with well–established evidence among non–Latino populations. Our findings suggests that the use of mindfulness training and emotional regulation skills through Cognitive Behavioral Therapy, Solution Focused Therapy, among others are the preferred and most reported approaches within an interdisciplinary sample of mental health professionals in Puerto Rico.

### Limitations

Although this study provided important descriptive and exploratory information regarding treatments used by an interdisciplinary sample of mental health professionals in Puerto Rico, where there exists a wide gap in knowledge and research on this topic, some limitations exist. First, the study findings were self–reported and included a convenience sample, which limited our ability to generalize the findings to mental health professionals across all of Puerto Rico. For example, a majority of participants graduated from private institutions, a group with greater attitudes of divergence towards research than those graduating from public institutions [21]. Second, study findings did not examine the use of these practices in combination with each other (e.g., integrative approaches), which is a growing area of interest, particularly when working with individuals at the intersection of social and economic vulnerabilities. Third, it is important to note that the self–reported use of these treatments does not translate to actual and appropriate use, especially if the intervention is incorrectly applied to a problem for which the practice does not have empirical support. For example, Cognitive Behavioral Therapy may be a well–established treatment for depression, but if a clinician is working with a client who has dissatisfaction with work, then its status as a well–established treatment might not be relevant. In this study, we used three available clearinghouses to vet EBTs given that the National Registry of Evidence–Based Programs and Practices (NREPP) was unavailable. In response to this methodological limitation, there is a need for greater investment in clearinghouses with rigor and breadth on what does or does not constitute an EBT [55]. Fourth, it is important to highlight that the EBPAS scale had limited response variability and appeared to be positively skewed. However, knowledge and training appeared to be strongly associated with the majority of treatments with well–established evidence. Future studies could explore whether attitudes are also favorable among a larger sample and whether they are related to more EBP behaviors. Fifth, survey responses were collected both online and in person. Unfortunately, we were unable to differentiate between participants who completed the survey online versus in–person to determine if differences in response patterns exist. Lastly, we acknowledge that the mental health professionals included in this study have varying levels of training and various specializations. We were unable to determine if these differences contributed to variations in the utilization of EBTs or in training, knowledge, and attitudes toward EBPs. Future research should explore how variations in training and specialization may impact the use of EBTs. For example, it will be important to include and evaluate how other individuals providing mental health services (i.e., psychiatrists, nurses, community health workers, among others) are interacting with EBTs in multiple settings.

### Strengths

This study is innovative in that it gathers a multidisciplinary and diverse sample of mental health professionals in Puerto Rico, an often overlooked population. The present study also provides preliminary information on the use of EBTs among mental health professionals, which has the potential to guide future dissemination and implementation efforts seeking to increase access to effective psychological interventions on the island. Additional strengths of this study included the low missing data rates and the gathering of a larger sample than expected. This allowed us to conduct descriptive and regression analysis of variables of interest.

## Conclusion

The use of effective EBTs is needed to meet the growing population of Puerto Ricans with mental health and substance use disorders. One of the central findings of this research was that nearly two thirds of an interdisciplinary sample of Latinx mental health professionals reported using an intervention with well–established research evidence. More importantly, individual and organizational factors, such as greater knowledge, training, and employment by a private agency predicted greater use of both well–established and experimental interventions. Results suggest that although the EBTs are not yet widely implemented in their entirety, there are grounds for optimism about advancing the quality of mental health services and the increased utilization of research informed practices in Puerto Rico. However, further research is needed to understand how Latinx mental health providers engage in the identification, adaptation, and evaluation of interventions and approaches for diverse settings and professionals. Greater investment in local research infrastructure/capacity could also lead to the development of home grown EBTs that could improve accessibility to high quality mental health services in Puerto Rico [56]. Such research could inform the development of evidence–based models that are culturally adapted and attuned to the needs of this community.

## Supporting information

S1 Checklist Inclusivity in global research. (DOCX)
